# A cost-consequence analysis of adding pertuzumab to the neoadjuvant combination therapy in HER2-positive high-risk early breast cancer in Italy

**DOI:** 10.1016/j.breast.2023.08.005

**Published:** 2023-08-08

**Authors:** Alberto Zambelli, Marina Cazzaniga, Nicla La Verde, Elisabetta Munzone, Ippazio Cosimo Antonazzo, Lorenzo Giovanni Mantovani, Serena Di Cosimo, Anna Mancuso, Daniele Generali, Paolo Angelo Cortesi

**Affiliations:** a Department of Biomedical Sciences, Humanitas University, Pieve Emanuele, Milan, Italy; b Medical Oncology and Hematology Unit, Humanitas Cancer Center, IRCCS Humanitas Research Hospital, Rozzano, Milan, Italy; c Phase 1 Research Centre, ASST-Monza (MB), 20900, Monza, Italy; d School of Medicine and Surgery, University of Milano-Bicocca, 20900, Monza, Italy; e Department of Oncology, Sacco Hospital, ASST Fatebenefratelli Sacco, Milan, Italy; f Division of Medical Senology, European Institute of Oncology, IRCCS, Milan, Italy; g Research Centre on Public Health (CESP), University of Milano-Bicocca, Monza, Italy; h Department of Advanced Diagnostics, Fondazione IRCCS Istituto Nazionale dei Tumori, Milano, Italy; i Salute Donna ONLUS, Milan, Italy; j Department of Medicine, Surgery and Health Sciences, University of Trieste, 34127, Trieste, Italy; k Multidisciplinary Unit of Breast Pathology and Translational Research, ASST of Cremona Hospital, 26100, Cremona, Italy

**Keywords:** Breast cancer, Pertuzumab, Neoadjuvant, Italy, Cost-consequence analysis

## Abstract

**Introduction:**

Clinical trials confirmed the beneficial effects of adding pertuzumab (P) to the combination of trastuzumab-chemotherapy (TC) in the (neo)adjuvant setting of high-risk HER2-positive early breast cancer (HER2+BC). We evaluated the clinical, economic and societal impact of adding pertuzumab to neoadjuvant TC combination (TPC) in Italy.

**Methods:**

A cost-consequence analysis comparing TPC vs. TC was performed developing a cohort-based multi-state Markov model to estimate the clinical, societal and economic impact of the neoadjuvant therapy of TPC versus TC in HER2+BC at high-risk of recurrence. The model works on a cycle length of 1 month and 5-years-time horizon. Literature review-based data were used to populate the model. The following clinical and economic outcomes were estimated: cumulative incidence of loco-regional/distant recurrences, life of years and QALY and both direct and indirect costs (€). Finally, sensitivity analyses were performed.

**Results:**

TPC was associated with a 75,630 € saved of direct costs. Specifically, it was associated with an initial increase of treatment costs (+4.8%) followed by reduction of recurrence management cost (−20.4%). TPC was also associated with an indirect cost reduction of 1.40%, as well as decreased incidence of distant recurrence (−20.14%), days of work lost (−1.53%) and days lived with disability (−0.50%). Furthermore, TPC reported 10,47 QALY gained (+2.77%) compared to TC. The probability to achieve the pathological complete response (pCR) was the parameter that mostly affected the results in the sensitivity analysis.

**Conclusion:**

Our findings suggested that TPC combination could be a cost-saving option in patients with HER2+BC at high-risk of recurrence.

## Highlights

• TPC in the (neo)adjuvant setting is an effective treatment in high-risk HER2-positive early breast cancer.
• TPC in the (neo)adjuvant setting is reimbursed in many European countries but not in Italy.
• In our analysis, TPC was associated with a 124,956 € saved per 100 treated patients over 5-year time horizon compared to TC.
• TPC showed a reduction of distant recurrence (−20.14%), days of work lost (−1.53%) and days lived with disability (−0.50%).
• TPC showed a cost-saving profile in patients with HER2-positive early BC at high-risk of recurrence compared to TC.

## 1 Introduction

Breast cancer (BC) is the most frequent malignancy affecting women worldwide, with 2.26 million new cases leading to 684,996 deaths in 2020 [1]. In Italy, BC was responsible for 834,154 cases of cancer among women (23% of all cancer cases in female and male) and about 12,500 deaths in 2021 [2]. BC is a heterogeneous disease, usually classified according to the expression of hormonal receptors and of human epidermal growth factor receptor 2 (HER2). HER2-positive breast cancer accounts for approximately 15% of all breast cancer diagnoses and it is characterized by a poor prognosis in the absence of specific HER2-targeting therapies [[3], [4], [5]]. The availability of anti-HER2-targeting therapies has dramatically improved the survival of these patients, significantly reducing the risk of recurrence and death. Nowadays, several biological therapies entered the clinic, including the monoclonal antibodies (mAb) trastuzumab, pertuzumab, the tyrosine kinase inhibitor (TKI) neratinib, and the antibody drug conjugate (ADC) T-DM1 and are now available for the (neo)adjuvant treatment of HER2-positive early BC [[4], [5], [6]].

Notwithstanding the relevant advantages obtained through the combination of these anti-HER2 treatments, approximately 25–30% of HER2-positive early BC still recur [7,8] and new approaches are needed to improve the clinical outcome of the patients. Currently, most patients with operable high-risk HER2-positive early BC (cT2-4 and/or cN+) receive neoadjuvant treatments [9]. The neoadjuvant approach allows the *in vivo* testing of treatment activity and eventually enables a personalized subsequent adjuvant therapy, providing significant survival advantage. The neoadjuvant regimen of antracycline/taxane chemotherapy plus trastuzumab/pertuzumab is the gold standard treatment for high-risk HER2-positive early BC [[10], [11], [12]] according to the evidence-based clinical research. Indeed, the seminal NEOSPHERE trial demonstrated that the addition of pertuzumab to trastuzumab (and chemotherapy) significantly increased the rate of pathological complete response (pCR) [[13], [14], [15]], in women with HER2-positive, locally advanced, inflammatory, or early early BC [9]. Similarly, different clinical trials confirmed the beneficial effects of the combination of trastuzumab and pertuzumab plus chemotherapy in the (neo)adjuvant setting [11,12], clearly defining the crucial role of pertuzumab in HER2-positive high-risk early BC.

Based on findings, the European Society of Medical Oncology (ESMO) recommendations stated the addition of pertuzumab to trastuzumab and chemotherapy as the optimal neoadjuvant approach in HER2-positive high-risk early BC [9] and in 2015 the European Medicine Agency (EMA) approved this combination in the neoadjuvant setting of patients with HER2-positive, locally advanced, inflammatory, early BC at high risk of recurrence.

Notwithstanding this approval, some European countries, including Italy, decided not to reimburse pertuzumab in the neoadjuvant setting [16], deriving patients disparity and questioning the universal access to the optimal BC care in Europe.

This study aimed to evaluate the clinical, economic and societal impact of adding pertuzumab to trastuzumab and chemotherapy in HER2-positive early BC patients, in order to provide additional information to help decision makers on the role of pertuzumab and its reimbursement in Italy.

## 2 Methods

### 2.1 Model overview

A cohort-based multi-state Markov model was developed to conduct a cost-consequence analysis in order to estimate the clinical, societal and economic impact of the neoadjuvant therapy of pertuzumab, trastuzumab plus chemotherapy (TPC) *versus* trastuzumab plus chemotherapy (TC) in HER2-positive early BC at high risk of recurrence. The methodological approach was based on a previous decision model health economic evaluations of neoadjuvant/adjuvant treatments in HER2-positive early BC patients and on the “Consolidated Health Economic Evaluation Reporting Standards (CHEERS) reporting guidelines” [[17], [18], [19], [20]]. The Markov model is a statistical approach to simulate different events or health states that individuals might experience. In a Markov model, each health states should be mutually exclusive and exhaustive, and the rate at which transition between health states occurs is called transition probability [21]. This statistical method allows for the incorporation of a repeated set of outcomes, and the transition matrix employs transition probability for individuals to move from one health state to another, considering associated costs and utilities [22]. For these reasons, it represents a valuable approach to perform health economic evaluations. To populate the model, data on effectiveness, safety, utility, and costs were gathered from clinical trials, literature review, and regional tariff guidelines.

**Fig. 1:**
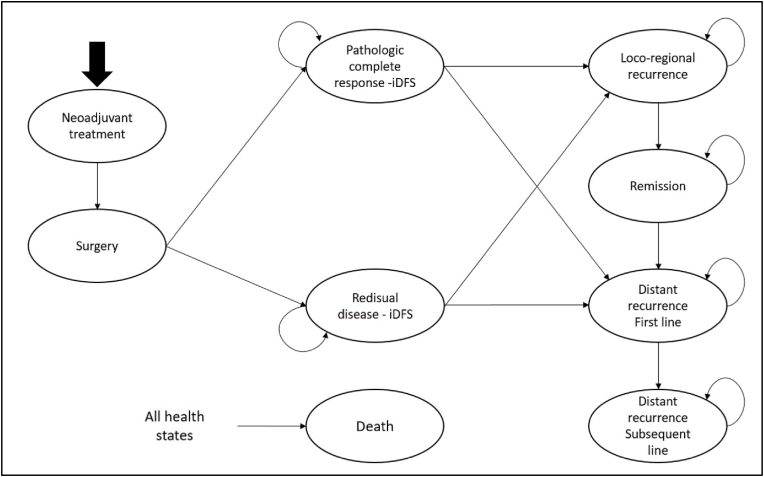
Markov Model structure.

The transition model simulated the movement of a hypothetical cohort of patients through 9 health states: 1) neoadjuvant treatment; 2) surgery; 3) pathological complete response (pCR); 4) residual disease (RD); 5) BC loco-regional recurrence; 6) remission; 7) distant recurrence with a first line treatment for metastatic BC; 8) distant recurrence with second and subsequent lines of treatment for metastatic BC; and 9) death (Fig. 1). The Markov cycle length was 1 month. All patients began the simulation in the neoadjuvant treatment status. During each monthly cycle, patients remained in the neoadjuvant treatment status and then transitioned to surgery. After surgery, the patients could report a pCR or residual disease (RD) with predictable different impact on the risk of recurrences. Accordingly, patients with loco-regional recurrence could transit to the status called loco-regional recurrence and then to remission; however, in this health status, the patients still continued to be at risk to develop distant recurrences. Patients developing a distant recurrence moved to the health status called “distant recurrence with first line of treatment”. Patients eventually failing the treatment in the “distant recurrence with first line of treatment” moved to the “distant recurrence with second and subsequent treatment lines”. Patients could transit to the “death” health status from all other conditions based on the individual and competitive probability of death. Movements of patients from a living state to death was based on the general population mortality and BC specific mortality occurring a distant recurrence. The model used a 5-years-time horizon and society point of view.

### 2.2 Population

The model simulated a population of 100 treatment-naïve women with HER2-positive early BC at high-risk of recurrence including clinical stages 2 and 3. The simulated cohort had a mean age of 50 years, a body weight of 71.5 Kg, a height of 163.10 cm, and body surface of 1.77 m2 (Table 1) [10].

**Table 1:** Key model inputs.

Parameter	Value^a^ (95%CI or Range)	References
**Clinical input**		
Age	50 (40–60)	[29]
Body weight (Kg)	71.42 (57.42–85.42)	[29]
Height (cm)	163.10 (131.13–195.07)	[29]
Body surface (m2)	1.77 (1.42–2.12)	[29]
Probability to achieve the pCR status under TPC treatment	0.61 (0.49–0.72)	[27,28]
Probability to achieve the pCR status under TC treatment	0.38 (0.31–0.46)	[29]
Monthly probability of distant recurrence in pCR patients	0.0003 (0.0002–0.0004)	[30,31]
Monthly probability of distant recurrence in pCR patients	0.0009 (0.0007–0.0010)	[31]
Monthly probability of loco-regional recurrence in RD patients	0.0003 (0.0002–0.0004)	[30,31]
Monthly probability of distant recurrence in RD patients	0.0028 (0.0022–0.0033)	[18,30]
Monthly probability of distant recurrence in patients with cancer remission	0.0017 (0.0014–0.0021)	[32]
Probability to switch from first line of pharmacological treatment to the other ones in patients with distant BC recurrence	0.0312 (0.0251–0.0373)	[33]
Probability of death in patients with distant BC recurrence under first line of treatment	0.0016 (0.0013–0.0019)	[33]
Probability of death in patients with distant BC recurrence under second or subsequent treatment strategies	0.0180 (0.0145–0.0215)	[23]
**Costs input**		
*Direct costs*		
Cost of TPC (4 cycles)	€ 0.43	[[39], [40], [41]]
Pertuzumab € per mg	€ 4.80	[[39], [40], [41]]
Trastuzumab emtansine (TDM-1) € per mg	€ 11.57	[[39], [40], [41]]
Paclitaxel € per mg	€ 2.53	[[39], [40], [41]]
Administration cost	€ 44	[40,41]
Surgery	€ 3.098.00 (2490.79–3705.21)	[40,41]
Monthly costs for management of patients under neoadjuvant therapy	€ 333.42 (268.07–398.77)	[35]
Monthly costs for management of patients under adjuvant therapy	€ 333.42 (268.07–398.77)	[35]
Monthly costs for management of patients free of recurrence	€ 114.42 (91.99–136.84)	[35]
Monthly costs for management of patients with loco-regional recurrence	€ 591.58 (475.63–707.53)	[35]
Monthly costs for management of patients in remission	€ 114.42 (91.99–136.84)	[35]
Monthly costs for management of patients with distant recurrence and under first line of treatment	€ 868.79 (698.51–1039.07)	[35]
Monthly costs for management of patients with distant recurrence and under second or subsequent treatment strategies	€ 1146.00 (1146.00–1146.00)	[35]
Monthly costs for management of patients with distant recurrence and with subsequent therapeutic strategy after the failure of TDM-1	€ 1635.38 (1314.84–1955.91)	[24,39]
*Indirectcosts*		
Number of monthly work days lost due to post-surgery treatment	10.42 (8.38–12.46)	[36]
Number of monthly work days lost in patiewnts free of recurrence	3.76 (3.02–4.50)	[36]
Number of monthly work days lost due to loco-regional recurrence	10.42 (8.38–12.46)	[36]
Number of monthly work days lost in patients with BC remission	3.76 (3.02–4.50)	[36]
Number of monthly work days lost due to distant recurrence under first line of treatment	14.51 (11.67–17.35)	[36]
Number of monthly work days lost due to distant recurrence under second/subsequent treatment strategy	14.51 (11.67–17.35)	[36]
Number of monthly work days lost due to surgery	22.44 (18.04–26.84)	[36]
Cost associated with a work day lost	€ 90.00 (72.36–107.64)	[34,36]
Number of monthly days lived with impairment in routine activities during post-surgery treatment	10.35 (10.28–10.42)	[36]
Number of monthly days lived with impairment in routine activities in patients free of recurrence	8.40 (8.35–8.45)	[36]
Number of monthly days lived with impairment in routine activities in patients with loco-regional recurrence	10.35 (10.28–10.42)	[36]
Number of monthly days lived with impairment in routine activities in patients in remission	8.40 (8.35–8.45)	[36]
Number of monthly days lived with impairment in routine activities in patients with distant recurrence under first line of treatment	14.64 (14.55–14.73)	[36]
Number of monthly days lived with impairment in routine activities in patients with distant recurrence under second/subsequent treatment strategies	14.64 (14.55–14.73)	[36]
Number of monthly days lived with impairment in routine activities due to surgery	30.44 (30.24–30.63)	[36]
**Utility inputs**		
Utility during neadjuvant treatment	0.829 (0.667–0.991)	[20]
Utility during post-surgery treatment	0.829 (0.667–0.991)	[20]
Utility of states free of recurrence	0.840 (0.675–1.005)	[20]
Utility of loco-regional recurrence	0.720 (0.579–0.861)	[18]
Utility of remission state	0.840 (0.675–1.005)	[20]
Utility of distant recurrence and first line of treatment	0.715 (0.575–0.855)	[18,37]
Utility of distant recurrence and second/subsequent treatment strategies	0.443 (0.356–0.530)	[18,37]
Disutility associated with surgery	−0.025 (0.20–0.030)	[38]

a Mean for number of days, costs, and utilities; probability for pCR and RD and for health state transitions (health state transitions probability are estimated from rates reported in the studies).

### 2.3 Treatment patterns

In the model, two treatment strategies were compared. Strategy 1 - TPC: patients received as neoadjuvant four cycles of trastuzumab (8 mg/kg loading dose followed by 6 mg/kg every 3 weeks), pertuzumab (840 mg loading dose followed by 420 mg maintenance every 3 weeks) and paclitaxel (80 mg/m2 every 12 weeks). Strategy 2 – TC: patients received as neoadjuvant four cycles of trastuzumab and paclitaxel with the same dosage and schedule. After surgery patients in both strategies received adjuvant treatment with trastuzumab (6 mg/kg every 3 weeks) or trastuzumab emtansine (TDM-1) (3.6 mg/kg every 3 weeks) based on the pathological response status, pCR versus residual disease, respectively, accordingly to the ESMO Guidelines [9]. Along with either strategy with trastuzumab or strategy with TDM-1 patients received the adjuvant treatment based on anthracycline-cyclophosphamide combination (Fig. 1 A supplementary material).

The model included specific treatment patterns for loco-regional and distant recurrence states. In particular, for loco-regional recurrence, patients were treated with TPC whereas, for distant recurrence, the patients were treated with TPC as first line- and with TDM-1 as second line of treatment. The duration of TDM-1 was set at 10 months as reported in the EMILIA trial [23]. Then, after the treatment with TDM-1 patients were considered treated with different antineoplastic treatments (i.e. control arm of the DESTINY BREAST 02 and/or HER2CLIMB clinical trials) [24,25].

### 2.4 Clinical data

Available data on the general survival surrogacy of established endpoints (i.e., pCR and RD) [26] were imputed in the model as proxy of neoadjuvant treatments' effect. Specifically, the model was populated with data from pivotal clinical trials on the studied treatment. In particular, in HER2-positive early BC the antitumor drugs activity was estimated by weighted the percentage of pCR both in the BERENICE and TRYPHAENA trials (TPC: 60.6%) [27,28] as well as in NOAH trial (TC: 38.5%) [29]. After neoadjuvant strategy, patients who achieved pCR were considered at lower risk of distant recurrence than those with RD. The model assumed a 15.9% 3-years risk of distant recurrence in patients with RD, as reported in the KATHERINE study. This risk is reduced by 40% in order to account the adjuvant TDM-1 efficacy [18,30]. Patients who achieved the pCR the 5-years risk of distant recurrence was 5% as reported by Symmans and colleagues [31]. The risk of loco-regional recurrence for pCR and RD patients was considered similar in both strategies based on Symmans's study and KATHERINE trial [30,31], and previous neoadjuvant treatment health economic evaluation [18]. Furthermore, for patients with loco-regional recurrence, the model applied a 10-years risk of distant recurrence of 18.9% [18,32]. Efficacy of first line treatment of distant recurrence were estimated from CLEOPATRA trial [33] with a treatment failure of 3.12% per month. The CLEOPATRA trial was used also to estimate the risk of death due to BC in this group of patients [33]. As reported in the EMILIA trial, the model assumed a risk of death of 2.11% for patients who began a second or subsequent line of treatment [23]. In addition, epidemiological data on the Italian general population mortality rate was retrieved by using the National Institute of Statistics – Istituto Nazionale di Statistica (ISTAT) [34]. These data stratified by age, was used to account in the simulation for risk of death due to other causes rather than cancer.

### 2.5 Cost input

The study was conducted adopting the societal perspectives; therefore, the direct and indirect costs were considered in the model (Table 1). The treatment costs were estimated based on the ex-factory price and undisclosed discount in the Lombardy region. The drug administration costs were based on the regional price tariff named MAC01. For the surgery procedure, the model included the mean costs associated with the hospitalization for different BC surgeries as indicated in the Lombardy regional tariff. Finally, an Italian study on BC costs was used to estimate the costs associated with the management of patients across the different tumour stages included in the model [35].

The model included also the indirect costs associated with BC: loss of productivity and daily life impaired by disease. The days of work-lost as well as the days with daily activity impaired were estimated by stage of disease based on Verrill and colleagues’ data [36]. The indirect costs associated with the number of productivity days-lost was calculated by multiplying the number of work days-lost due to disease, during the simulated period, with the average wage (90 euros per day) reported by the Italian general population statistics-ISTAT [34].

### 2.6 Health states utility input

Patients in the event-free state were assumed to have better quality of life (QoL) than those in the recurrence states. Health state utilities were obtained from data available in the literature [[18], [19], [20],^37^]. The utility associated to each health state was used to estimate the quality-adjusted life per years (QALY) for each of the compared treatment strategy. As reported in previous studies, for patients who received surgery, was applied a disutility of −0.02487 to account in the model for the impact of this procedure on the QoL of the patients (Table 1) [20,38].

### 2.7 Outcome

In this cost-consequence analysis, for each of the included treatment option, the main economic, clinical and societal outcomes were estimated. Specifically, as economic outcomes the model estimated the direct and indirect costs associated with the two treatment strategies. The model estimated the direct costs of the neoadjuvant and adjuvant therapy; these included: the cost of therapies; the drug administration costs; the costs associated with the management of patients with loco-regional recurrence as well as those associated with distant recurrence and under different line of treatments. For the estimation of indirect costs, the model included those related to the productivity lost. As regards, the clinical outcomes, the model estimated: cumulative incidence of loco-regional recurrences; cumulative incidence of distant recurrences, life of years and QALY. Finally, from the societal perspective the model estimated: loss of productivity (days of work-lost) and impact of disease on routine activities (number of days in which the patients experienced impairment in routine activities).

### 2.8 Statistical analysis

The cost-consequence analysis compared the direct and indirect costs and the consequence (clinical and societal outcomes) of TPC neoadjuvant therapy with the standard of care with TC. The results were expressed as mean value and delta difference per 100 treated patients.

To test the robustness of the analysis, a sensitivity analysis was performed by using a time-horizon of 10 years. In addition, the impact of model parameters variability was assessed via one-way sensitivity analysis. Specifically, we iteratively replaced individuals model input by using their 95% confidence intervals or alternatively high and low value of range (calculated by using mean and standard error) and re-estimated model results, holding other inputs constant. Finally, we rank-order the resulting set of sensitivity analysis by absolute magnitude of deviation from the base case to assess which input parameters mostly affected the results. The one-way sensitivity analysis was applied to test the parameters variability on overall costs, cumulative incidence of distant recurrences, QALY and daily activities/loss of productivity (days of work lost and number of days in which the patients experienced impairment in daily activities).

## 3 Results

In our simulation, the use of TPC (strategy 1) as neoadjuvant therapy compared with the use of TC (strategy 2) was associated with a reduction of costs in 5-years simulation. In particular, the use of TPC was associated with 124,966 euros saved per 100 treated patients (Table 2). As reported in Table 2, the use of the TPC was associated with an initial increase of treatments cost of about 4.8% (3,397,847 euros for TPC Vs 3,241,629 for TC per 100 treated patients) and with a parallel reduction of about 20.4% of costs for the management and treatment of distant recurrence (938.791 euros for TPC Vs 1.178.800 euros for TC per 100 treated patients). The analysis of indirect costs showed similar results to the previous one. Overall, the TPC was associated with a reduction of 1.40% (8,818,992 euros for TPC Vs 8,943,948 euros for TC per 100 treated patients) of total costs (Table 2).

**Table 2:** Cost-consequence analysis results. Results are reported as outcomes and costs per 100 treated patients.

	Neoadjuvant TPC	Neoadjuvant TC	Difference TPC vs TC	Delta (%)
***Economic outcomes***				
Costs of neoadjuvant-adjuvant therapy	3,397,847 €	3,241,629 €	156,218 €	4.82%
Costs of management of patients free of recurrences	821,573 €	814,242 €	7330.67 €	0.90%
Cost for treatment of distant recurrence	68,760 €	68,052 €	708.36 €	1.04%
Cost of management of loco-regional recurrence	10,243 €	10,139 €	104.13 €	1.03%
Cost of management of patients in remission	3095.90 €	3078 €	18.29 €	0.59%
Cost of therapy for distant recurrence	734,095 €	921,658 €	−187,563 €	−20.35%
Cost of management of patients with distant recurrence	204,696 €	257,142 €	−52,446 €	−20.40%
Total direct costs	5,649,965 €	5,725,595 €	−75,630 €	−1.32%
Costs associated with productivity lost	3,169,027 €	3,218,353 €	−49,326 €	−1.53%
Total costs	8,818,992 €	8,943,948 €	−124,956 €	−1.40%
***Clinical outcomes***				
Cumulative incidence of loco-regional recurrences	1.61	1.59	0.018	1.14%
Cumulative incidence of distant recurrences	8.32	10.42	−2.10	−20.14%
Life years	495.22	494.69	0.53	0.11%
QALY	388.66	378.19	10.47	2.77%
***Societal Outcome***				
Number of days of work lost	35,211	35,760	−548	−1.53%
Number of days lived with routine activities impairment	55,999	56,282	−283	−0.50%

The favourable economic profile of TPC compared with TC was also associate to clinical and societal benefits. In fact, the TPC was associated with a numerically decreased of cumulative incidence of distant recurrences (delta: 20.14%) and number of days of work lost (delta %: 1,53%). Additionally, TPC therapy showed a reduction in the number of days lived with disability (difference: 283 days per 100 treated patients; delta %: 0.50%). Further, in 5 years of simulation, TPC produced 388.66 QALY compared with 378.19 QALY produced by TC with 10,47 QALY gained (delta %: +2.77%) per 100 treated patients.

Findings from the sensitivity analysis performed by using a longer time-horizon (10 years) were in line with those reported in the based-case scenario. In particular, the use of neoadjuvant TPC showed a higher value as indicated by economic and clinical outcomes improvement compared with the TC strategy. In particular, in a 10-years simulation, TPC was associated with 673,822 euros saved compared with treatment with TC. Additionally, TPC treatment was also associated with a numerically decreased incidence of distant recurrences (4 events per 100 patient) and increase of QALY 27.27 per 100 treated patients.

**Fig. 2:**
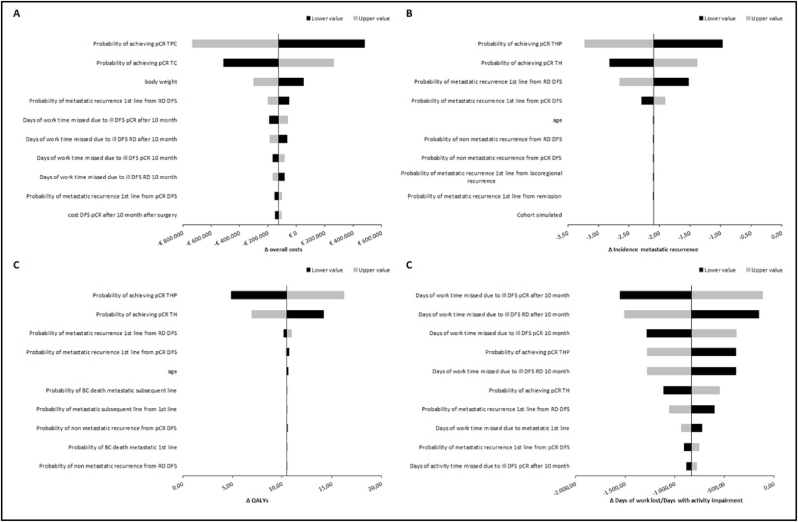
Tornado diagram for one-way sensitivity analysis on the following outcomes: a) Overall costs, b) Incidence of metastatic recurrence, c) QALY gained and d) days of work lost/days with activities impairment.

The one-way sensitivity analysis confirmed the good cost-consequence profile of TPC compared with TC. In the OWSA, the probability to achieve the pCR was the parameter that mostly affected the overall costs, incidence of metastatic recurrence and QALY gained. Days of work lost and days of daily activities impairment were mainly affected by the number of day lost or impaired associated to patients with pCR and free from recurrence (Fig. 2).

## 4 Discussion

Since 2015 the use of TPC is authorized in Europe as neoadjuvant therapy in case of HER2-positive, locally advanced, inflammatory, early stage BC with a high risk of recurrence. Nonetheless, in Italy this treatment is still not reimbursed by the NHS, therefore this study, for the first time, attempts the cost-consequence associated with the TPC treatment compared with TC, which is the current standard of care in the country. The aim of the study was focused on a model-based economic evaluation; it was performed to examine the cost-consequence of neoadjuvant TPC *versus* TC, adopting the Italian societal perspective. Findings from this study showed that the use of TCP reduce the 5-years overall costs compared to TC. The TPC combination is also associated with an improvement of clinical and societal outcomes. In particular, TPC is associated with a reduction of days of work lost due to progressive disease, because of the estimated reduction in the rate of distant recurrences and improved prognosis with an increase of QALY [9,42,43]. In addition, the estimated higher rate of pCR with use of neadjuvant TPC will derive a limited use of T-DM1, as escalating treatment in the adjuvant setting, with a substantial benefit for the quality of life of the patients [44].

The efficacy and the favourable safety profile of pertuzumab in the neoadjuvant setting, has already been assessed in several real-world observations in other authorized markets and eventually should be confirmed in the Italian national context. However, the apparent additional costs associated with new treatments forced the regulatory agencies to perform proper health economic analyses to assess the real benefits and price/value of these drugs to ensure the optimal NHS resource allocation. All together the aforementioned type of data are crucial to guarantee sustainable access to high-value care for patients, in particular for patients with cancer.

Although pertuzumab as neoadjuvant therapy is not really a novelty, the evidence of its value and cost is still limited. Our findings were in line with those reported in previous published studies [20,45,46]. Borges and colleagues found that the addition of pertuzumab as neoadjuvant therapy was associated with an increase of 40% of the overall costs per patient and an improvement of clinical outcomes (i.e., pCR response) [45]. However, it should be noted that this was a real-world study including a small cohort of patients. Our study, corroborates the positive impact of pertuzumab/trastuzumab as neoadjuvant therapy by exploring the impact of these medications on multiple outcomes such as indirect costs and different clinical outcomes. The cost-effectiveness favourable profile of neoadjuvant pertuzumab/trastuzumab combination was observed also in a Canadian study [46] in which the authors performed two cost-utility analyses by using the data from the NeoSphere and TRYPHAENA data. Findings suggested that based on the NeoSphere data, the combination resulted in an incremental cost per life year gained of 23,658 dollars and per QALY of 25,388, values that slightly increased, reaching values of 43,000 dollars per LY and 46,000 per QALY when the data from the TRYPHAENA trial was included in the model [46]. Sussel et al., developed a hybrid decision tree with Markov state-transition simulation model to compare several competing neoadjuvant/adjuvant treatment strategies for high-risk HER-2 positive BC patients by using data from the US health system [20]. Findings from the aforementioned study suggested that strategies including neoadjuvant pertuzumab/trastuzumab combination yielded the highest overall clinical benefits. Neoadjuvant pertuzumab/trastuzumab approach was less costly and more effective (dominant) compare to only trastuzumab [20]. Similar results were found also in another model-based economic evaluation that compared five (neo)adjuvant treatments for patients with HER2 positive early BC [18]. The authors showed that paclitaxel along with the use of trastuzumab/pertuzumab followed by adjuvant dose-dense anthracycline/cyclophosphamide and T-DM1 in case of RD or only trastuzumab for those who obtained the pCR was a cost-effective treatment associated with highest health-related benefits and lowest costs compared with other alternatives in the same setting [18]. However, the aforementioned studies adopted different study protocols and designs, and therefore a direct comparison with our findings is not worthy. From above, we may conclude that adding pertuzumab to the neoadjuvant TC treatment combination in HER2-positive high-risk early BC, improved the patients clinical outcome along with a not negligible costs reduction. Our findings confirm the previous data from extra-European studies, by adding relevant evidence of the direct and indirect treatment costs as well as the clinical and societal outcomes, from an European country.

Even if we attempted to perform the most robust study and provide the most reliable results, our study has some limitations that need to be discussed. Firstly, we needed to make assumptions to fully specify the model in order to translate the complex medical decisions and treatments pathways in the model used for the analysis. In our approach, we assumed that the effectiveness of studied treatments in real-world setting were similar to the treatment efficacy showed in the clinical trials. In this context, it is well known that difference in patient's outcomes might exist between clinical trials and real-world evidence studies [47]. These differences might stem from different factors, including differences in patient's characteristics, treatment approaches, and health care practice. Real world studies might have limitations in comparing different treatment approaches due to the possible bias associated to observational studies, further the differences in effects reported in real world studies is an issue related to all treatment investigated with a possible different efficacy reported by each treatment. However, despite these limitations, post-marketing phase, real-world data can be valuable in supporting policymakers in periodically reassessing the drug's effectiveness and value after regulatory approval. For this reason, there should be collaboration between clinicians, pharmaceutical companies, and healthcare policy makers to conduct real-world evidence studies, which might be used to assess the prediction provided by decision model on treatment effectiveness and its impact on patients and the healthcare system [47,48]. In addition, we showed the pooled results for all patients, without stratification by nodal status or other relevant clinical factors. Thirdly, the costs associated with mastectomy and/or conservative surgery plus irradiation treatment were not included in the model. Further, it should be noted that, recently, a subcutaneous formulation, of a fixed-dose combination of trastuzumab and pertuzuamb has been approved by the US food and Drug administration (US-FDA) and European Medicine Agency (EMA) [49]. This new formulation has the potential to favourably impact on the clinical daily management of HER2-positive early BC patients and represents an important opportunity for a less invasive, faster and safer drug administration with expected time-saving benefits and side-effects reduction, including catheter-associated discomfort, thrombosis and infection [[50], [51], [52], [53], [54]]. In this perspective, the current general cost-consequence model deserves to be regularly updated. In the era with the availability of new effective treatments for BC, updated pharmaco-economic studies are crucial to assess the economic value of the therapeutic alternatives and to estimate their cost-effectiveness in order to properly allocate the economic resource and guarantee the optimal treatment option to the patients.

## 5 Conclusion

The combination of trastumab/pertuzumab and chemotherapoy (TPC) represents the gold standard treatment in patients with HER2-positive early BC at high-risk of recurrence, according to all national/international guidelines. For the first time, our findings suggest that adding pertuzumab to neoadjuvant regimen is an effective and cost-saving approach with a favourable clinical, economic and societal impact in the Italian national context. The results of this cost-consequence analysis might inform policy-makers to plan public health intervention in order to guarantee an universal patients access to optimal and sustainable treatment, reaffirming the role of the Italian NHS in ensuring adequate levels of life-saving care. Further studies are needed to confirm our findings in real world clinical practice in Italy and in other countries.

## Author contributions

All authors contributed to conceive or design the work. All authors contributed to the acquisition, analysis and interpretation of data, and drafted and critically revised the manuscript. All authors gave final approval and agree to be accountable for all aspects of the work, ensuring integrity and accuracy of the manuscript.

## Funding

The study was supported by an unrestricted grant by 10.13039/100004337Roche S.p.A.

## Declaration of competing interest

AZ, MC, NLV, EM, ICA, SDC, AM, DG have no conflicts of interest to disclose. DG has received Honoraria from Novartis, Lilly, Roche, Pfizer, Istituto Gentili. Research funding from profit organizations as Astrazeneca, 10.13039/100004336Novartis and Myriad. PAC has received a research grant from 10.13039/100009431Baxalta now part of Shire, and speaking honoraria from 10.13039/100004319Pfizer, 10.13039/100004337Roche and Novartis. LGM has received grants and personal fees from 10.13039/100004326Bayer AG, Boehringer Ingelheim, 10.13039/100004319Pfizer and 10.13039/501100002973Daiichi-Sankyo. MN is employes of Novartis Pharma AG.
