# Supplementary material for: A cost-consequence analysis of adding pertuzumab to the neoadjuvant combination therapy in HER2-positive high-risk early breast cancer in Italy
Source: Breast. 2023 Aug 8;71:113–21. doi: 10.1016/j.breast.2023.08.005 (PMC10428118; doi:10.1016/j.breast.2023.08.005)
Supplement: Multimedia component 1 [file mmc1.docx]

Figure A1. Treatment strategies (neoadjuvant+adjuvant) included in the model.


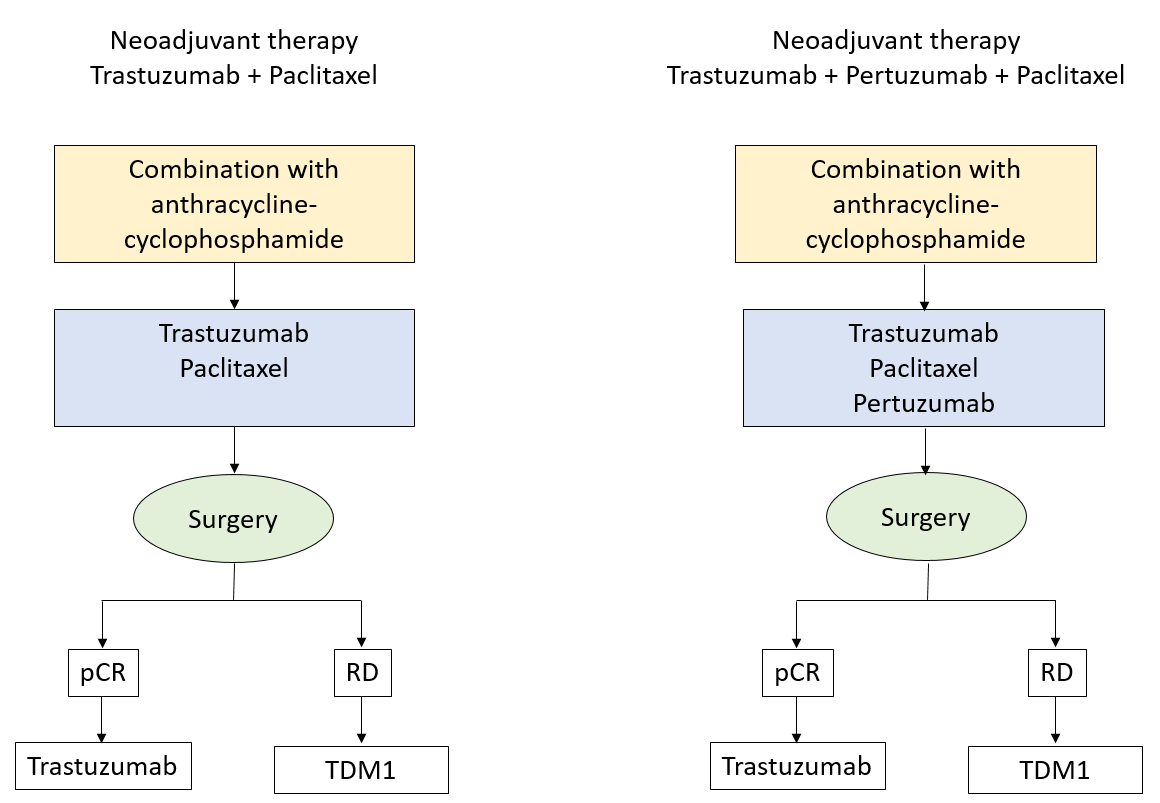


Legend: pCR: pathological complete response; RD: residual disease
